# Benchmarking MedViT and hybrid CNN–ViT architectures for multi-label thoracic disease classification

**DOI:** 10.1038/s41598-026-43282-5

**Published:** 2026-04-10

**Authors:** Victor Agbo, Ruchi Patel, Munindra Lunagaria, Lokesh Malviya, Akshay Jadhav, Sandeep Monga

**Affiliations:** 1 Department of Computer Engineering, Marwadi University, Rajkot, Gujarat India; 2 School of Computing Science & Engineering, VIT Bhopal University, Sehore, M.P. India; 3 School of Computer Science and Engineering, Manipal University Jaipur, Jaipur, Rajasthan India

**Keywords:** Vision transformer (ViT), Chest X-ray (CXR), Multi-label classification, Medical image analysis, Deep learning, Computational biology and bioinformatics, Diseases, Health care, Mathematics and computing, Medical research

## Abstract

Computer-aided diagnosis relies heavily on the automatic classification of thoracic diseases from chest X-ray (CXR) images, yet this task remains challenging due to class imbalance, overlapping radiological features, and high inter-class similarity. In this study, two architectures MedViT and Hybrid CNN–ViT are adapted and evaluated, which are a scalable Vision Transformer (ViT)-based architecture designed for multi-label thoracic disease classification. MedViT is enhanced with transfer learning, domain-specific augmentations, and self-attention mechanisms to capture subtle pathological patterns across diverse conditions. The Hybrid CNN–ViT is the combination of strength of CNN and ViT which is admirable in capturing local patterns. Both models are trained and validated on two benchmark datasets, NIH ChestX-ray14 and CheXpert, and compared against state-of-the-art baselines. On the NIH ChestX-ray14 dataset, MedViT showed strong performance with 93.34% accuracy and a macro AUROC of 94.17%, while the Hybrid CNN–ViT model reached 85.81% accuracy and 72.28% macro AUROC. On the CheXpert dataset, MedViT achieved 79.22% accuracy and a macro AUROC of 75.11%, whereas Hybrid CNN–ViT achieved 76.15% accuracy and 71.68% macro AUROC. These results show that MedViT performs well and generalizes effectively across different datasets. Per-label analysis demonstrated robust precision and recall even for under-represented conditions such as fibrosis and hernia, where existing models typically show significant performance drops. Unlike earlier methods that often struggle with generalization, MedViT maintains a balanced trade-off between sensitivity and specificity across all categories. These findings highlight the effectiveness of Transformer-based feature encoding in capturing subtle spatial correlations in medical imaging, while also setting new benchmarks for automated thoracic disease classification. The MedViT model outperformed the state-of-the-art methods and shows strong potential to support radiologists in decision-making and improve diagnostic workflows in clinical practice.

## Introduction

Lung diseases constitute a substantial share of the global disease burden. WHO data show 8 million deaths every year worldwide attributed to respiratory diseases like chronic obstructive pulmonary disease (COPD), pneumonia, tuberculosis, and lung cancer^1^. In the year alone 2019, COPD became a prime factor in causing about 3.2 million deaths, becoming the third leading cause of deaths worldwide^2^, with lower respiratory infections generally remaining the most deadly communicable disease group among children and older adults^3^. Besides these, lung cancer causes 1.8 million deaths globally each year, thereby making it appear at the top of the list of cancer-causing deaths^4^. The economic cost is also staggering: it is about €380 billion per year on direct and indirect costs of healthcare in the states of the European Union^5^. Considering that there are about 2 billion people currently exposed to household air pollution while smoking is still prevalent in many parts, respiratory diseases would have a higher incidence^6^. The above statistics should stimulate action in developing countries, especially those where scant access to radiologists and advanced diagnostics would warrant early detection and appropriate management strategies.

These diseases consistently cause a lot of morbidity and mortality for people in all age brackets^7^. Acute progressive diseases like pneumonia, asthma, COPD, tuberculosis, pulmonary fibrosis, or lung cancer cause malfunctioning of the respiratory system, less lung capacity, poorer gas exchange, and occasionally, respiratory failure^8^. Most of these diseases share or exhibit overlapping symptoms like cough, fever, and chest discomfort, making an early and accurate diagnosis basing simply on the clinical presentation very difficult^9^. Chest radiography is a core part of the diagnostic system under pulmonary medicine^10^. Although it is inexpensive, quick, and capable of justifying visual evidence of infiltrates, nodules, consolidations, and pleural effusions, it is the highly used method among clinicians^10^. Though effective, the interpretation of the images received is very much dependent on the expertise with which that is done. This is not available to most areas of the world, due to trained radiologists becoming increasingly rare^11^, and this has become a very serious hurdle in receiving timely diagnosis and care.

AI, mainly deep learning, is a growing ally for solving diagnostic problems in medical imaging today^12^. Convolutional neural networks (CNNs) have been very successful in classifying diseases in image data, including applications in dermatology^13^, ophthalmology^14^, and radiology. In the realm of thoracic imaging, various deep learning models have been trained to identify conditions such as pneumonia, pneumothorax, and COVID-19, with performance levels competitive to or even surpassing human experts^15^. However, most of the existing algorithms are severely constrained by their design to single-label classification, allowing identification of only one pathology per image. While this is a massive limitation in itself, multiple lung diseases usually occur in tandem, especially in the elderly or immunocompromised individuals. Therefore, Multi-label classification of several diseases on a single radiograph is a more clinically relevant and useful approach. Making robust models that can handle such complexity is an active area of research that could greatly improve diagnostic accuracy, clinician workload, and expert-level interpretation in resource-limited environments.

Even though diagnostic systems are fast improving, they fail to grasp the intricacies that rank full in real-world clinical situations relating to lung diseases. Most current deep-learning approaches are restricted to single-label classification, which means they would detect one disease per image either way while multiple coexisting conditions exist^16^. This holds clinical significance since several patients, generally the elderly or those having compromised immunity, can have two or more concurrent pulmonary ailments. Additionally, label imbalance in datasets presents a restriction due to the inadequate representation of infrequently occurring diseases; the lesser the representation, the more unreliable and less generalizable the model becomes. Interpretability and scalability issues arise with models trained on narrow datasets, and these usually show poor performance across diverse clinical settings. To advance diagnostic accuracy and boost clinical practice, there is now increasing demand for a more spheric and adaptive approach: one that should be capable of multi-label classification, utilize data augmentation to counter label imbalance, and ensure robustness across heterogeneous data sources. Such an approach would reflect the clinical reality better and help provide broader yet more precise support to healthcare professionals, especially in settings lacking radiological expertise.

Several important metrics were used to evaluate the overall performance of the proposed model for multi-label chest X-ray classification, including binary accuracy, which measures the correctness of predictions for each label individually. Additionally, Precision, Recall, and F1 Score were employed to provide a more holistic view of the model’s capability to correctly identify pathologies while minimizing false positives and false negatives. The evaluation of model discriminative capability also took place at various thresholds following the AUC-ROC. For training, the loss function was Binary Cross-Entropy (BCE), which is a good fit in the context of multi-label classification architectures. BCE is well equipped to deal with discerning each label independently as a binary prediction since multiple labels may coexist conditionally within an image. It also ensures that the treatment of disease labels remains weight-balanced relative to the application of any subsequent alterations of the dataset introduced to address label imbalance.

The primary contribution of this study to the evaluate the adaptation and enhancement of multi-label classification of thoracic pathologies using a deep learning model, MedViT and Hybrid CNN-ViT. The long-range dependency and contextual feature capturing ability of MedViT, a vision transformer architecture optimized for medical imaging, made it a natural choice for evaluating radiographic images. For robust training of the model and offering improved performance for underrepresented labels, the NIH ChestX-ray14 and CheXpert data sets were rebalanced by duplicating cases in the minority labels and adjusting label-specific loss weights, thus alleviating the effects of label imbalances without introducing any synthetic data. Both data sets were used to quantify the generalizability and consistency of MedViT across different clinical settings. The obtained results were analyzed using standard classification metrics, which gave a comprehensive understanding of the diagnostic utility of the model in the real world. Besides MedViT, this paper also experimented a Hybrid CNN-ViT combination, which integrates the strength of CNNs in the local extraction of features with that of vision transformers in the global representation. The architecture allows the model to learn fine-grained lesion details and at the same time learn long-range dependencies in X-rays of the chest. The comparison between MedViT and the Hybrid CNNViT allows the study to gain a better insight into the performance of the various transformer-based designs in the context of multi-label medical imaging tasks.

### Research goals

- Develop a robust multi-label classification system capable of detecting multiple lung pathologies from a single chest X-ray.
- Compare the performance of MedViT and a custom hybrid CNN-ViT model on large-scale medical imaging datasets.
- Evaluate generalizability and consistency by testing both models on two benchmark datasets: NIH ChestX-ray14 and CheXpert.
- Assess classification performance using key metrics such as Accuracy, Precision, Recall, F1-Score, and AUC.
- Address the challenge of coexisting pathologies in thoracic imaging by leveraging advanced deep learning architectures.
- Contribute to resource-limited settings by proposing models that reduce dependency on expert radiological interpretation.

### Research questions

- How well does MedViT and a hybrid CNN-ViT model classify multi-label lung disease from chest X-rays?
- Does the performance of these models generalize well across datasets such as NIH ChestX-ray14 and CheXpert?
- Which one showed better performance across evaluation metrics such as Accuracy, Precision, Recall, F1-Score, and AUC?
- Are multi-label classification approaches better in the diagnostic utility of chest radiographs than single-label methods?
- How much does an AI-based diagnostic tool help in reducing the dependence on radiologists, especially in low-resource environments?

### Research contributions

- Built and tested a multi-label framework for lung disease classification, setting MedViT beside a hybrid CNN–Vision Transformer and measuring what each could bear under the same conditions.
- Worked carefully with the NIH ChestX-ray14 and CheXpert datasets, aligning labels, separating patients, and carrying models across datasets to see what findings held true and what failed.
- Compared the models methodically, using accuracy, precision, recall, F1-score, and AUC to mark their strengths and their limits.
- Addressed the reality that disease does not arrive alone, adopting a multi-label strategy to reflect the overlapping pathologies found in clinical practice.
- Explored how automated interpretation of chest X-rays might lessen delay and ease the burden on radiologists, offering a quieter, faster companion to clinical judgment.

The remainder of this article is organized as follows: Section “Literature review” presents the related work on thoracic disease classification using various deep learning methods. Section  “Research methodology” describes the methodology to apply MedViT and the Hybrid CNN–ViT models on two different datasets. Section “Experimental evaluation” outlines the experimental setup and evaluation metrics. Section  “Discussion” reports and discusses the results obtained on NIH ChestX-ray14 and CheXpert datasets. Finally, Section “Conclusion” concludes the study with key findings and directions for future research.

## Literature review

As times go on, the attention towards data quality, generalization of the model, and hybrid architecture is increasing. The advances in deep learning have improved performance; however, issues like label imbalance, few annotations, and domain variability still exist. Newer strategies are being devised to tackle them by better utilization of data, transfer learning, and integration of CNN s and relevant transformer-based models. Table 1 presents a consolidated summary of prior research on Thoracic Disease Classification, while the subsequent subsections provide a detailed method-wise review of the existing approaches.

### Data-centric approaches

For a long time, computer-aided diagnosis (CAD) systems have worked on a model-centric approach through which the aim was to refine the neural network architecture, modify the number of layers, and optimize the loss functions. Though these have improved the work, model improvements often perceive less importance of another vital factor—the training data regarding its quality and characteristics. In recent years, however, the shift toward data-centric methodologies is rather obvious; which rather focus on bringing about improvement to model’s output through curation, enrichment, and structuring of datasets employed for training, rather than modifying solely the model^17^.

Nguyen et al. (2022) provide one of the clear examples following such trend in which the authors recommended the data-centric deep-learning method for the better detection of pulmonary nodules on CT images^18^. Instead of redesigning model architectures from scratch, the authors concentrated on enhancing the dataset quality. They analyzed several properties of the intrinsic data such as nodule sizes and nodule aspect ratios to find great imbalances in the widely adopted LUNA16 dataset. To correct for this, they added a new dataset-referred to as the K dataset-culled from Vietnamese clinical sources, composed of 382 CT scans and 170 annotated nodules. These nodules had different size distributions compared to LUNA16, thus providing some much-needed variability.

By combining the LUNA16 and K datasets into a more diverse and balanced LUNA16 + K dataset, and by fine-tuning anchor box configurations in object detection models to match the revised data characteristics, they observed improved detection sensitivity. The enhancements were evident across three popular detection frameworks: YOLOv3, RetinaNet, and Faster R-CNN, with sensitivity improvements of up to 4.24%. This study highlights a fundamental insight in modern AI applications for healthcare: refining and tailoring the training data can lead to performance gains that are sometimes more significant than those achieved by modifying model architecture alone. Building on this perspective, our study similarly adopts a data-centric philosophy. Instead of relying solely on advanced generative augmentation techniques or oversampling via GANs, we focus on balancing underrepresented disease classes through straightforward yet effective strategies such as duplication and class weighting.

### Class imbalance in other medical image tasks

Class imbalances remain among the spectrum of core challenges that tend to recur in the segmentation tasks of medical images - especially in using most current machine and deep learning models for medical images. An imbalanced dataset usually contains classes that bear a majority number of representations by pixels concerning the tissue backgrounds or healthy anatomical structures, while only a small number of representations would be present for regions of clinical interest such as tumors, lesions, or pathological tissues. Most real-world databases have this not incidental but intrinsic to the nature of the medical image itself; for instance, in brain MRI or CT images, histopathological tissues might occupy only 1% of the volume in which the majority (99%) consists of healthy brain tissue or other structures around it. As highlighted by Shahbazi et al.^19^, models tend to become biased toward the majority class and produce predictions with high confidence for a background classification that altogether misclassifies the minority class, which usually contains most of the information.

In segmentation tasks, this bias can significantly impair the model’s ability to generalize and learn from the pathological features. Since learning algorithms are typically optimized to minimize overall loss, they may achieve this objective by simply focusing on accurately predicting the abundant majority class, while errors in the minority class contribute relatively little to the total loss. As a result, evaluation metrics such as overall pixel-wise accuracy may appear deceptively high, giving the false impression that the model is performing well. This performance gap underscores the importance of using more informative evaluation metrics that account for class imbalance.

Dice score, sensitivity (true positive rate), and specificity (true negative rate) are much more appropriate metrics in instances where the detection of rare but vital regions in images is important^20^. These measures emphasize the accurate minority class identification, penalizing false negatives more prominently, an element of great importance in medical diagnosis where missing a lesion can lead to severe clinical ramifications. In cancer screening tasks, a very minimal dip in sensitivity arguably implies undiagnosed cases from which treatments are not administered soon enough.

To deal with these, a plethora of techniques have been put forth based on both data level and algorithm level strategies. Algorithm wise, different uses of weighed loss functions, weighted cross-entropy, and focal loss that impose heavier penalties on the misclassification of the minority class are among the avenues that researchers have explored. Yet another popular option includes patch-based training in which input patches are sampled more from ‘minority’ class regions so that the model really has seen enough examples of rare pathological features during training^21^. Hybrid solutions that combine - such as synthetic data augmentation through generative models (GANs), class rebalancing, or curriculum learning - have also shown promise in counteracting the effects of imbalance.

Addressing class imbalance, however, is not just a technical optimization effort but also foundational for creating reliable segmentation systems that are trustworthy and deployable in the clinic. If this aspect is not confronted, it will lead to systems that look good on paper but ultimately fail in application. This therefore calls for the careful incorporation of imbalance-aware metrics, training strategies, and evaluation procedures in the design of segmentation pipelines so that the performance of AI models can be made robust, fair, and clinically meaningful to both major and minor classes.

### Transfer learning and pretrained models

Transfer learning has become the holy grail in medical image segmentation when dealing with scarcity or high cost of available annotated datasets. Instead of training models from scratch, for which massive amounts of labeled data and computation would be needed, transfer learning harnesses knowledge from models trained previously on large datasets such as ImageNet. This, in turn, quickens convergence, reduces overfitting, and promotes generalization—all the more enticing for healthcare applications where diversity of data and labelling expertise are often limited.

In a recent example, Chanhoe Gu et al. systematically evaluated the performance of different pretrained convolutional neural networks (CNNs), including ResNet18, ResNet50, ShuffleNet, and Inception-v3, for pneumonia detection in chest X-rays. The reports found constant enhancement in performance after the implementation of transfer learning. For instance, ResNet18 training from scratch gave a test accuracy of 89.6%. The use of pretrained weights raised it by a sizeable margin to 93.1%. Inception-v3 gained further increase in precision, recall, and F1-score, thus proving the value of pretrained representations in drawing relevant features from complicated medical images^22^.

Keeping further complexity in mind, studies carried out by G. Ayana et al. examined multistage transfer learning. This transfer learning entails sequential transfers of learned representations from one domain into another domain, which enables the models to adapt better across varied modalities, imaging protocols, or clinical environments. The study found models pretrained on a diverse collection of datasets to be better able to withstand domain shifts, including variances in scanner types, patient demographics, and acquisition settings. The model robustness is necessary to achieve the generalization of the models in real-time deployment, where the training and target data distributions often show a significant difference^23^.

Equally promising in this area is self-supervised learning (SSL): the pretrained models with unlabeled data to learn meaningful features through proxy tasks—such as image inpainting, rotation prediction, or contrastive learning. Kalapos and Gyires-Tóth (2022) demonstrated that self-supervised pre-training on unlabeled images specific to the medical domain produced an exhilarating 4–5× acceleration in convergence as opposed to the conventional methodologies. Astonishingly, even given fine-tuning on small labeled datasets, the self-supervised models performed at par with the ImageNet-pretrained networks. This approach provides a very enticing solution to the annotation bottleneck that persists in many clinical applications^24^.

Collectively, these studies emphasize three crucial advantages of leveraging transfer learning in medical image analysis:

- Faster convergence and better generalization by transferring low- and mid-level features from natural images to medical tasks^22^,
- Improved robustness and domain adaptation through multistage transfer across diverse clinical environments^23^,
- Label-efficient learning by harnessing unlabeled data in self-supervised pretraining pipelines^24^.

Building upon these insights, our work introduces a hybrid architecture that integrates DenseNet-121 with a Vision Transformer (ViT). DenseNet-121 is established through dense connectivity in reuse of features and is very good at capturing the local spatial patterns in a medical image, while the global context modeling is being derived through self-attention mechanisms by ViT. Hence, this combination is meant to balance very fine detailed local understanding with the overall understanding of the image—both being critical in the medical diagnosis process. Both components of DenseNet and ViT are initialized using ImageNet-pretrained weights to harness the well-known feature hierarchies, and thus this hybrid method leads to accelerated convergence and better performance on two extensive medical image datasets: NIH and CheXpert Chest X-rays, even though labeled examples are much less in that learning from them. The pretrained architecture takes advantage of knowledge complementarity to bridge between inadequate data and clinical credibility.

### Traditional CNN approaches

Convolutional Neural Networks (CNNs) are developed to serve as the backbone for medical image segmentation due to their potential in extracting hierarchical features as well as capturing essential local spatial patterns for anatomical structure and pathological region identification. The U-Net architecture with the encoder–decoder design and skip connections is verified to perform well across a range of tasks for tumor, organ, and lesion segmentation. Improved variations are implemented, such as U-Net++  and Attention U-Net, which add dense skip connections and attention mechanisms, respectively, for better refinement of features and also better focus on areas of concern. DenseNet-121 is a very good architecture for the construction of a DNN to mitigate the vanishing gradients and help stabilize its training and also generalize better with the small number of medical datasets by its densely connected layers^25^.

While CNN-based architectures have become the de facto choice for many medical imaging tasks, they are not without shortcomings. Their fundamental design principles such as centered on local receptive fields and fixed convolution kernels—impose constraints when applied to the complex and varied nature of medical images. Moreover, the diversity in imaging modalities, patient populations, and clinical environments further exposes the limitations of traditional CNNs. As medical imaging demands a high level of precision and adaptability, understanding these challenges is crucial to motivating more advanced or hybrid modeling strategies.

Despite their strengths, CNNs face critical limitations in the context of medical imaging:

1. Limited receptive field – Standard convolutional layers struggle to capture global context, which is often essential for segmenting anatomically distant but clinically related regions.
2. High dependency on labeled data – CNNs require extensive annotated datasets, a challenge in medical domains where expert labeling is costly and time-consuming^26^.
3. Restricted adaptability and interpretability – CNNs are sensitive to domain shifts (e.g., scanner differences) and their black-box nature limits clinical trust^27^.

Due to these apparent limitations, researchers have had to develop new architectures that were hybrids of CNNs with transformer-based components. The hybrid architectural model marries the local feature extraction strength of CNNs with global attention mechanisms provided by transformers. Therefore, it handles spatial specificity and contextual awareness. So far, the evidence from the application of hybrid architectures in clinical segmentation tasks-cases like multi-organ CT segmentation or lesion detection has shown better robustness, generalization to unseen data, and higher interpretability in comparison to each network alone. Hybridization, therefore, emerges as a great candidate for next-generation medical AI systems.

### Vision transformers

Vision Transformers or ViTs have arisen as a strong candidate against Convolutional Neural Networks (CNNs) in the field of computer vision, including in medical image analysis. The CNNs rely on local filters to extract local patterns, while ViTs utilize self-attention methods to model global relations between image patches and to ponder much longer-range dependencies, which may be particularly advantageous in medical imaging scenarios where the contextual information could be across the entire image such as when differentiating between diffuse patterns of a disease or looking for small lesions against a complex anatomical background.

In architectures based on ViT, an image is divided into fixed-size patches, embedded linearly, and augmented with positional encodings before being treated in the transformer layers. This allows the ViTs to attend globally to regions that are spatially distant but semantically related in a single pass. Dosovitskiy et al. first introduced the ViT model pretrained on ImageNet and demonstrated that, given sufficient data, it can outperform CNNs on various vision benchmarks^28^. Inspired by this, medical imaging researchers have adapted ViTs to domain-specific tasks. For instance, Hatamizadeh et al. proposed UNETR, a hybrid model combining transformers with a U-Net-like decoder, and showed substantial improvements on 3D volumetric segmentation tasks^29^. Similarly, Chen et al.‘s TransUNet integrated ViTs into a CNN framework for organ segmentation and achieved better generalization than traditional CNNs, particularly in cross-domain evaluations^30^.

While ViTs provide significant advantages, they are not without limitations. Their performance is heavily dependent on large-scale pretraining and sufficient fine-tuning data. In low-data regimes common in medical imaging, ViTs may underperform unless adapted with strategies such as self-supervised pretraining, data augmentation, or hybrid modeling with CNNs. Additionally, their computational demands due to the quadratic complexity of self-attention which can be challenging in volumetric or high-resolution 3D imaging.

To overcome these issues, our approach integrates ViT modules with a DenseNet-121 backbone, creating a hybrid model that combines CNNs’ efficiency in local feature extraction with transformers’ strength in global context modeling. This architecture capitalizes on pretrained ViT encoders to improve classification accuracy on the NIH and CheXpert datasets, particularly for subtle or spatially dispersed abnormalities. The hybrid design not only accelerates convergence but also enhances robustness to variations in anatomy and imaging conditions.

### Multitask and attention-based models

Multitask learning (MTL) and attention mechanisms have gained considerable traction in medical image segmentation due to their ability to improve generalization and enhance focus on diagnostically relevant features. These strategies address core challenges such as label scarcity, noisy annotations, and inter-class similarity by explicitly guiding the network to learn complementary tasks or to prioritize relevant regions during training.

Multitask models train a shared backbone to perform multiple related tasks simultaneously for example, lesion segmentation, classification, and anatomical landmark detection. This shared representation encourages the network to learn richer, more generalizable features. A notable example is Peng et al.’s work on multitasks learning for cardiac MR segmentation and disease classification, which showed that auxiliary tasks improved the main segmentation performance by enforcing anatomical consistency and promoting robust feature extraction^31^. Similarly, Wu et al. introduced a model that jointly learned polyp segmentation and edge detection, showing superior boundary preservation and higher Dice scores in colonoscopy datasets^32^.

In parallel, attention mechanisms particularly spatial and channel attention have been widely adopted to help models focus on the most informative parts of medical images. Attention gates, as used in Attention U-Net, dynamically suppress irrelevant background features and highlight salient regions, leading to improved precision in segmenting small or diffuse abnormalities. Wang et al. demonstrated that attention modules in a hybrid CNN-transformer architecture significantly enhanced lung and liver tumor segmentation by refining focus on ambiguous or low-contrast areas^33^.

Combining MTL with attention mechanisms has proven especially effective. These models benefit from the complementary strengths of both approaches: MTL enforces holistic feature learning across tasks, while attention modules refine spatial localization. However, one limitation is that multitask frameworks require carefully curated auxiliary labels, and improper task selection may lead to negative transfer. Similarly, attention mechanisms introduce additional computational overhead and may require tuning to balance sensitivity and specificity across classes.

In our work, we incorporate attention layers within a hybrid DenseNet–ViT architecture to enhance focus on disease-relevant regions. The DenseNet and ViT branches extract complementary features, which are concatenated and used for multi-label classification on NIH ChestX-ray and CheXpert datasets.

### Ensemble and dual model frameworks

Ensemble and dual-model frameworks have been gaining considerable popularity in medical image analysis for combining techniques to enhance diagnostic accuracy and robustness through multi-model or multi-network employability. Ensemble learning combines outputs from several base learners, frequently pretrained deep neural networks, to mitigate the biases and variances of the individual models. The heterogeneous feature representations extracted by different architectures brush the clutter off the complex classification task, such as multi-class detection of lung diseases from chest X-rays.

A recent example is the study by Patel and Shah (2024), who developed a deep ensemble learning model to classify four lung conditions: COVID-19, pneumonia, lung opacity, and normal states, using chest X-ray images^34^. Their approach concatenated features extracted from three pretrained CNNs (VGG16, InceptionV3, and MobileNetV2) followed by classification through machine learning classifiers such as support vector machines (SVM) with a majority voting scheme. This two-level ensemble model achieved a high classification accuracy of 93%, which further improved to 94% using a three-level ensemble strategy, demonstrating the effectiveness of feature fusion and ensemble classification in dealing with imbalanced and noisy datasets. Their methodology also included advanced preprocessing techniques and data resampling to address data quality and class imbalance, which are common challenges in medical imaging.

The dual-model method usually constructs two parallel networks of complementarity, capturing local fine-grain features from one side and global contextual one from the other side. For instance, hybrid architectures that combine CNN backbones with Vision Transformers (ViTs) are expected to meaningfully incorporate spatial details and long-range dependencies at the same time and attain better segmentation and classification results^29^, ^30^. These frameworks help overcome individual model limitations such as CNNs’ restricted receptive field or transformers’ data requirements.

Together, ensemble and dual model strategies represent effective avenues for enhancing the robustness and generalizability of medical image analysis systems, making them well-suited for clinical applications where diagnostic accuracy is critical.

Other modern approaches tackle semi-supervised segmentation. ERSR focuses on fetal head ultrasound with limited annotations. It filters predictions, refines pseudo-labels with ellipses, and enforces symmetry-based consistency. The result is reliable, shape-aware supervision^34^. PG-FANet addresses histopathology. It combines inter- and intra-uncertainty regularization with multi-scale, multi-stage feature aggregation. Even with few labels, it achieves state-of-the-art results^35^. IPA-CP targets tumor segmentation. It uses uncertainty-guided augmentation, iterative pseudo-label transitions, and bidirectional copy-paste supervision to handle small and multiple lesions effectively^36^.

**Table 1:** Summarized review of existing research.

References	Year	Technique/Model	Dataset(s)	Key results (with metrics)
^22^	2023	Transfer Learning (ResNet18, Inception-v3)	Pneumonia Chest X-rays	ResNet18: Accuracy ↑ from 89.6% to 93.1%; Inception-v3: F1-score ↑ from 87.2% to 91.5%
^23^	2024	Multistage Transfer Learning	Multiple medical sets	Improved generalization; AUC ↑ by 3–5% across domains
^24^	2022	Self-Supervised Pretraining	2D medical images	Dice coefficient ≈ 0.87 vs. 0.84 (ImageNet), convergence 4× faster
^25^	2022	DCSAU-Net (Split-Attention U-Net)	Private CT segmentation	Dice ↑ 3.2% vs. U-Net; Params ↓ 45%, training time ↓ 27%
^27^	2023	Review: CNNs vs. ViTs	Various segmentation	ViTs improve Dice by up to 5% in low-data settings; better global context modeling
^29^	2022	UNETR (Transformer-based 3D U-Net)	BTCV, MSD	Dice = 0.912 (liver), 0.867 (pancreas); ↑ 2.4% over 3D U-Net
^30^	2021	TransUNet (CNN + Transformer hybrid)	Synapse multi-organ CT	Mean Dice = 0.835; ↑ 2.9% vs. U-Net++
^33^	2023	TransBTS (Multimodal Transformer)	BraTS brain tumor MRI	Mean Dice = 0.894; Sensitivity = 0.882, Specificity = 0.917

## Research methodology

**Fig. 1:**
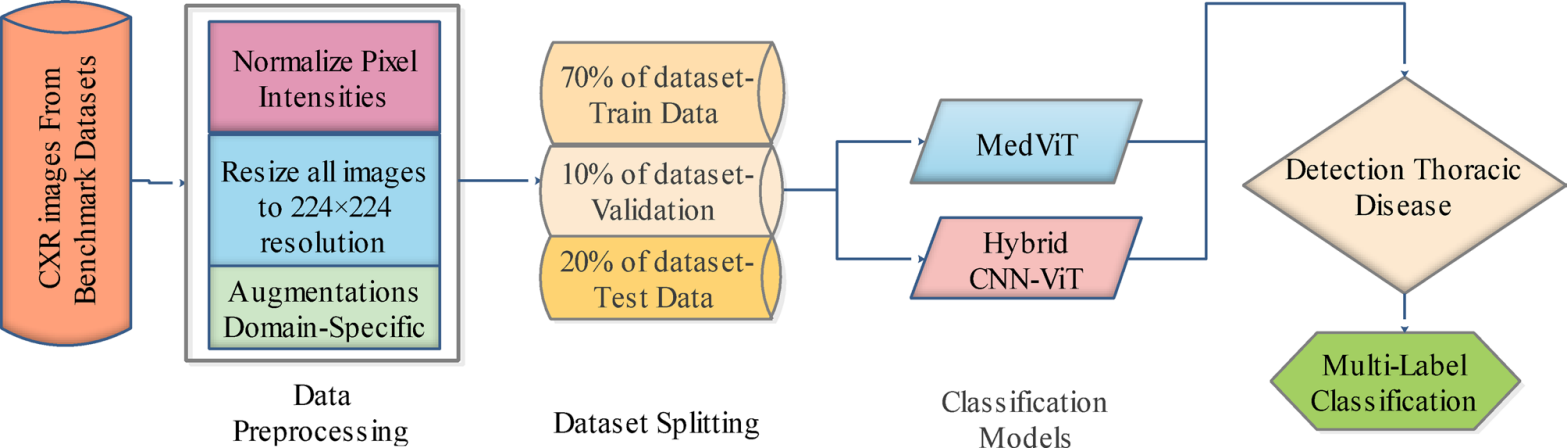
High level flowchart shows the steps taken in the project.

This section offers information on our approach to lung disease classification. A flowchart of the research technique is shown in Fig. 1.

### Dataset description

Chest X-ray (CXR) imaging remains a cornerstone in clinical diagnostics due to its non-invasive nature, widespread availability, low cost, and relatively low radiation exposure. It is often the first imaging modality used for evaluating thoracic abnormalities, particularly in high-volume clinical settings such as emergency departments and intensive care units. Leveraging the diagnostic utility of CXRs, large-scale public datasets have been developed to support the training and evaluation of deep learning models for automated disease classification. This study utilizes two publicly available, large-scale chest radiograph datasets: the NIH ChestX-ray14 and the CheXpert datasets. Both serve as important benchmarks for developing and evaluating automated medical imaging systems in thoracic disease classification.

#### NIH ChestX-ray14

**Fig. 2:**
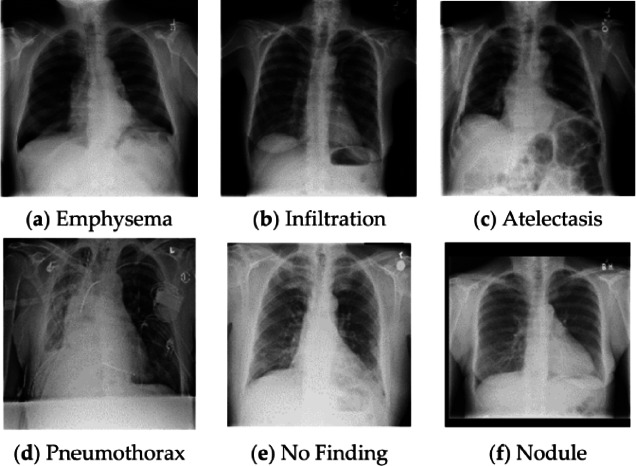
Sample images of NIH Chest X-ray 14 dataset^37^.

The NIH Clinical Center provided its ChestX-ray14 dataset, including 112,120 anterior-posterior (AP) frontal chest X-ray images collected from 30,805 unique patients. Sample images are shown in Fig. 2. The images are in PNG format and have been downsampled to the fixed resolution of 1024 × 1024 pixels. Each image is assigned to one or more of 14 thoracic pathology labels: Atelectasis, Cardiomegaly, Consolidation, Edema, Effusion, Emphysema, Fibrosis, Hernia, Infiltration, Mass, Nodule, Pleural Thickening, Pneumonia and Pneumothorax. The labels were defined from the corresponding radiology reports by a rule-based natural language processing (NLP) tool, which brings in some level of uncertainty since there are limitations in automated label extraction from free-text narratives.

#### CheXpert

There are 224,316 chest radiographs in the CheXpert dataset, which were acquired from 65,240 patients by the Stanford Machine Learning Group. Images are made in DICOM format, usually converted into PNG files, and resized around 320 by 320 or 224 by 224 pixels size to streamline studies. We selected 224 × 224 for both datasets as the size for our work, although the original DICOM files may have varying native resolutions. The dataset provides labels for 14 observations with the possibility to categorize findings as positive, negative, or uncertain. For this work, we will focus on a subset of seven pathology classes i.e. those labels that have an overlap with NIH’s dataset and are strictly thoracic diseases, corresponding to clear, discrete disease entities: Atelectasis, Cardiomegaly, Consolidation, Edema, Pneumonia, Pleural Effusion and Pneumothorax. This selection is based on their clinical significance and well-characterized radiographic manifestations.

In CheXpert, annotations marked as uncertain (− 1.0 in the metadata file) were treated as negative, and only findings explicitly labeled as positive were taken to indicate disease.

For this research, the data for the NIH ChestX-ray14 dataset was obtained from a CSV file that organizes into proper format the metadata and image annotations. This file contains essential information like image file paths, patient identifiers, and labels for diseases, which allow systematic data access and preprocessing. Each record corresponds to a single frontal chest radiograph and has multi-label annotations revealing that the image specimen suffers from one or more thoracic conditions. The dataset is split into three portions which are 70% training, 10% validation, and 20% test, while keeping the patient-wise splitting to avoid data leakage and improve generalizability. First, all unique patients were identified using their Patient IDs, and then the train, validation, and test sets were created based on those patients.

**Fig. 3:**
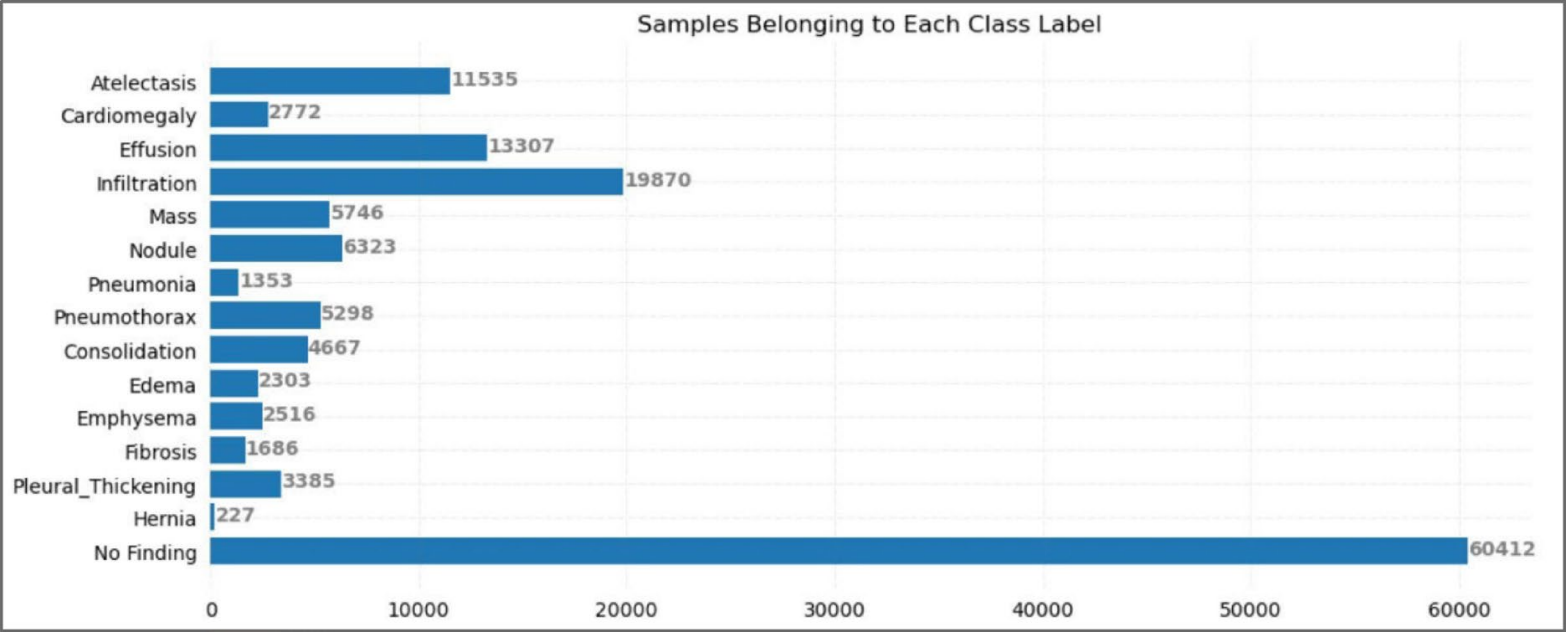
Class distribution in the NIH ChestX-ray14 dataset, showing significant imbalance—Infiltration has 19,870 images, while Hernia has only 227^37^.

However, a notable attribute of the dataset is its high degree of label imbalance - a common dilemma in medical imaging datasets, shown in Fig. 3. For instance, the Hernia class is very poorly represented, with just 227 images, whereas Infiltration is the class with the highest representation, with 19,870 images. This imbalance warrants some critical thinking in training the model, such as class weighting, oversampling after the patient-wise split, or specialized loss functions to reduce biased learning towards majority classes. To mitigate this, more weight was given to classes with lower representation.

Both datasets provide grayscale images, and preprocessing steps (including format conversion and resizing) were applied to ensure consistency across training and evaluation stages.

By leveraging the breadth of NIH ChestX-ray14 and the improved label fidelity of CheXpert, this study benefits from both dataset scale and diagnostic specificity.

### Dataset preprocessing and augmentation

In medical imaging, with special reference to chest X-ray investigation, data-preprocessing and augmentation are required keys to getting maximum performance out of the deep learning models. Preprocessing typically involves scaling or normalizing pixel values so that the model can more effectively learn its objective function with consistent input distributions. This is an operation of immense importance when the architecture relies on input variance, as in the case of Vision Transformers (ViTs), where input is processed in patches. When constructing the dataframe to be used by model, entries with the ‘No Finding’ label were completely removed, to prevent bias a huge portion of the dataset has that label as seen in Fig. 3. Also, this is to ensure focus in placed on the actual diseases and there is no bias in the model towards ‘No Finding’ in a clinical setting. This step was performed to reduce class imbalance by narrowing the disparity in sample counts, for example between No Finding (60,412 samples) and Infiltration (19,870 samples).

Augmentation in turn refers to the increase in the diversity of data artificially, by introducing controlled variations to the training images. The process of augmentation assists in counter fighting the problem of overfitting, especially for imbalanced datasets, while also forcing the model to learn variant and generalizable features. Common augmentation methods for the image data include:

- Flipping the image horizontally to simulate mirrored anatomical structures.
- Random brightness adjustments to mimic real-world radiographic conditions.
- Resizing and cropping to normalize spatial dimensions.
- Adding noise or slight distortions to account for sensor or acquisition variability.
- Applying geometric transformations such as slight rotations or shifts.

In this study, tailored augmentation techniques were selectively applied to chest X-ray images to preserve diagnostic features while enhancing the robustness of the MedViT model. These preprocessing and augmentation strategies collectively contribute to more effective training and improved generalization on unseen patient cases.

#### Image preprocessing

Crucially, preprocessing in a MedViT-based pipeline prepares chest X-ray images for proper training and successful feature extraction. All images were first loaded from disk in JPEG format, decoded, and resized with bilinear interpolation to 224 × 224 pixels. This resolution matches the input requirements of MedViT and other ViT-based architectures that operate on fixed-size image patches. Standardizing input dimensions is equally critical in obtaining consistent patch embeddings across the dataset.

The next step was to divide all intensity values by 255.0 to normalize pixel values so that they fell within the [0,1] range. This normalization is very important for ensuring numerical stability while training and achieving fast convergence by constraining the input distribution. Probably, there was no need for extra normalization in the form of mean subtraction and standard deviation scaling since the MedViT model is supposed to be immune to such changes based on its architecture and the patch-wise processing it implements.

Using the TensorFlow tf.data API for fast loading, transforming, and caching of image-label pairs, the dataset was pre-processed. These preprocessing steps were uniformly applied across the training, validation, and test splits, ensuring consistency and thus preventing any biases in favour of any dataset.

#### Image augmentation

To further enhance model generalization and reduce the risk of overfitting, data augmentation techniques were selectively applied to the training dataset. Given the structural sensitivity of vision transformers, particularly to spatial distortions, only light augmentations were used to preserve anatomical integrity while still introducing meaningful variability.

Specifically, two forms of augmentation were implemented:

- Random horizontal flipping, with a probability of 0.5, was used to simulate mirrored anatomical structures while retaining clinical plausibility.
- Random brightness adjustment, with a maximum delta of 0.1, introduced controlled illumination variability, mimicking real-world radiographic conditions.

These augmentations were implemented using TensorFlow’s image transformation functions and applied dynamically during batch creation, ensuring that each training epoch receives a slightly different view of the data. Importantly, no augmentation was applied to the validation or test datasets, to maintain the consistency and objectivity of performance evaluation.

The final data pipeline included shuffling with a large buffer (at 5000 samples per class) to ensure thorough label mixing, followed by batching and prefetching for optimal GPU/TPU utilization. This end-to-end data pipeline allowed the MedViT model to learn more robust features from the chest X-ray images while maintaining computational efficiency.

### Traning phase

This section outlines some of the key information related to the training process, including model types, hyperparameters, and architecture of classifiers.

#### Dataset split

Label imbalance and dataset partitioning are two of the most significant things to consider when training effective deep learning models with medical imaging data. For example, the variances in disease prevalence within the ChestX-ray14 dataset- where ‘Hernia’ has a mere 227 images but ‘Infiltration’ has over 19,000. Therefore, a label-wise resampling strategy was employed to ensure proper representation and eliminate bias during training. Each disease category was sampled to have at least 5,000 label instances, either through upsampling or data reuse, depending on the originally available images.

Since this is a multi-label multifaceted type of data where an image may represent multiple disease classes, the resultant number of label instances (i.e., disease-image pairs) would exceed the number of unique images. After balancing, the number of total label instances in the dataset was approximately 70,000.

For training and evaluation, the dataset was divided using a 70:10:20 ratio into training, validation, and testing subsets, respectively. This split was stratified to preserve class distributions across subsets. The patient-wise splitting is applied on dataset to avoid data leakage and improve generalizability. First, all unique patients were identified using their Patient IDs, and then the train, validation, and test sets were created based on those patients. Table 2 shows disease positive cases according to dataset split.

**Algorithm 1:**
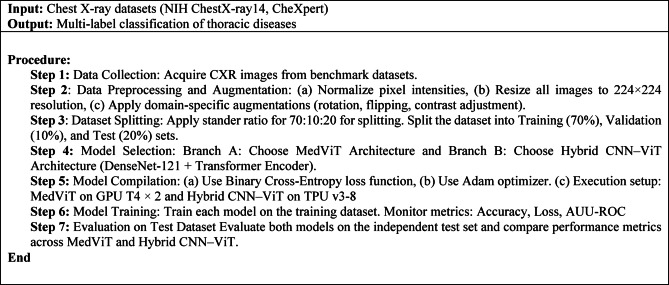
Workflow for MedViT and Hybrid CNN–ViT Training

- Total label instances: ~70,000.
- Estimated unique images: fewer than 70,000 (due to multi-label overlaps).
- Data allocation:
  - Training set: ~49,000 label instances (70%).
  - Validation set: ~7,000 label instances (10%).
  - Test set: ~14,000 label instances (20%).
- Unique image count:
  - Total count: 34,250.
  - Train: 24,271.
  - Val: 3538.
  - Test: 6441.

**Table 2:** Disease positive cases by dataset split.

Disease	Train_Pos	Val_Pos	Test_Pos
Atelectasis	5485	779	1468
Cardiomegaly	1712	272	515
Consolidation	2668	397	789
Edema	1522	200	436
Effusion	6393	914	1717
Emphysema	1562	256	519
Fibrosis	1131	182	325
Hernia	158	25	44
Infiltration	7437	1102	2073
Mass	3825	534	778
Nodule	3830	559	1088
Pleural_Thickening	2105	325	575
Pneumonia	977	150	273
Pneumothorax	3601	530	763

In addition to preventing overfitting, this partitioning technique was essential for guaranteeing an impartial and broadly applicable assessment of the model’s performance across all disease categories. The count of unique images after oversampling.

#### Model architecture

To effectively capture both local and global patterns present in chest radiographs, two distinct deep learning architectures were explored. The MedViT model served as the primary architecture due to its efficiency and domain-adapted vision transformer design. A secondary Hybrid CNN-ViT model was also implemented to evaluate the complementary benefit of convolutional inductive biases combined with global self-attention mechanisms.

##### MedViT architecture

**Fig. 4:**
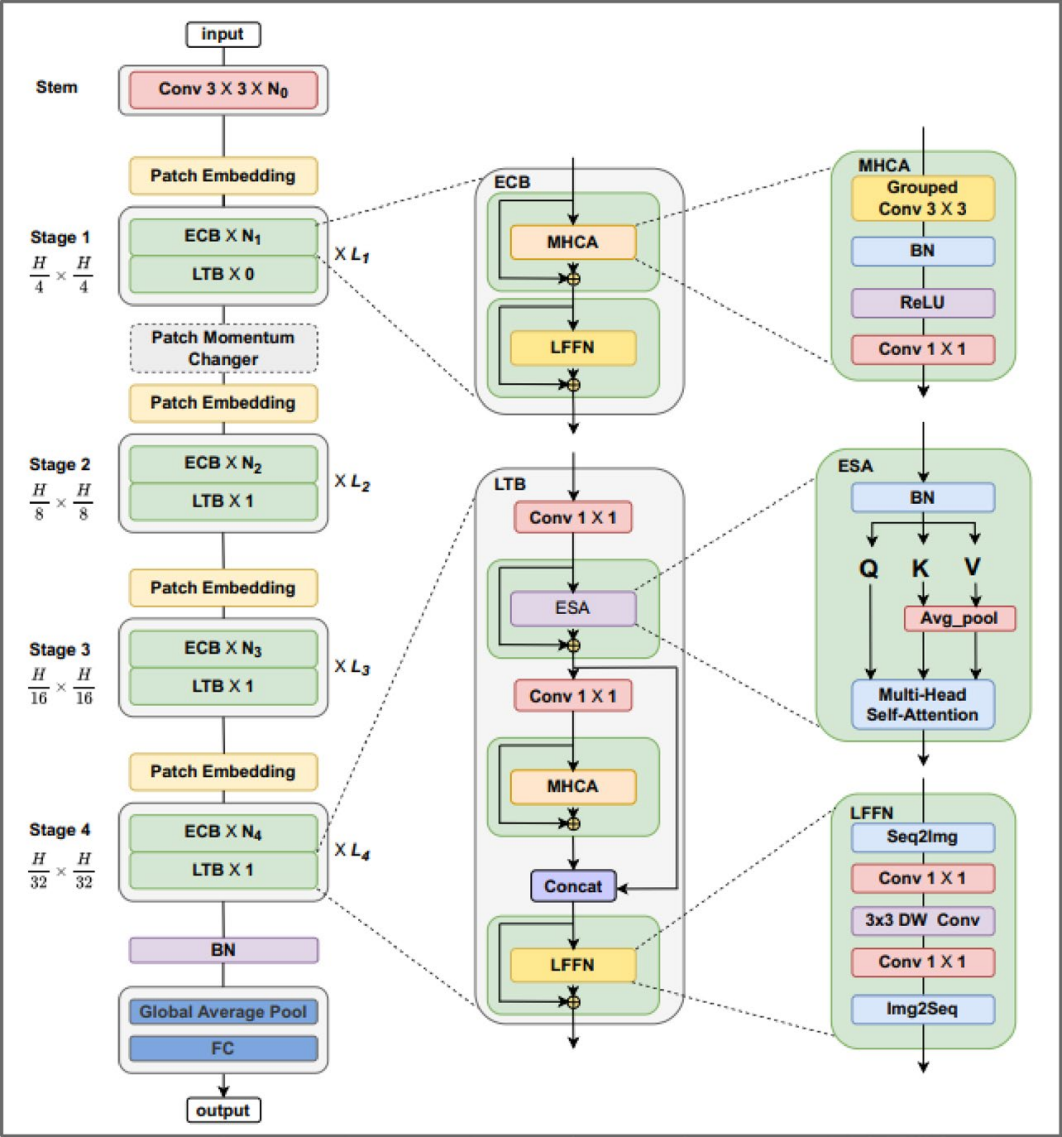
MedViT architecture for multi-label chest X-ray classification using patch embedding and transformer encoders^38^.

MedViT is a lightweight Vision Transformer architecture specifically tailored for medical imaging tasks. Unlike standard ViT models, which are computationally intensive and require large-scale pretraining, MedViT incorporates architectural refinements that make it more data-efficient and suitable for relatively smaller-scale medical datasets. The architectural diagram shown in Fig. 4.

The MedViT pipeline includes the following components:

- Patch Extraction: Each input image of size 224 × 224 is divided into non-overlapping 16 × 16 patches, resulting in a total of 196 patches per image.
- Patch Embedding and Encoding: Each patch is projected into a high-dimensional embedding space and augmented with positional encodings. A learnable class token is appended to capture global image context.
- Classification Head: A global average pooling layer aggregates token outputs, which are passed through a fully connected layer with 14 sigmoid activations—one for each disease class.

The ‘small’ variant of MedViT was selected for a balance of speed and accuracy. Its configuration is given below:

**Table:** 

Attribute	MedViT_small
Total parameters	31.1 M
Backbone layers	CNN stem + multiple ECB & LTB blocks
Embedding sizes	96 → 192 → 256 → 384 → 512 → 768 → 1024 (progressive in blocks)
Attention heads	MHCA/E_MHSA layers; typically 4–8 per block (check MHCA init in code)
Patch size	Patch embedding is 1 × 1 in each MHCA step;

This architecture enables the model-to-model long-range dependencies across anatomical regions in chest radiographs, a property that is critical for identifying diffuse or co-occurring disease pattern.

##### Hybrid CNN–ViT architecture

**Fig. 5:**
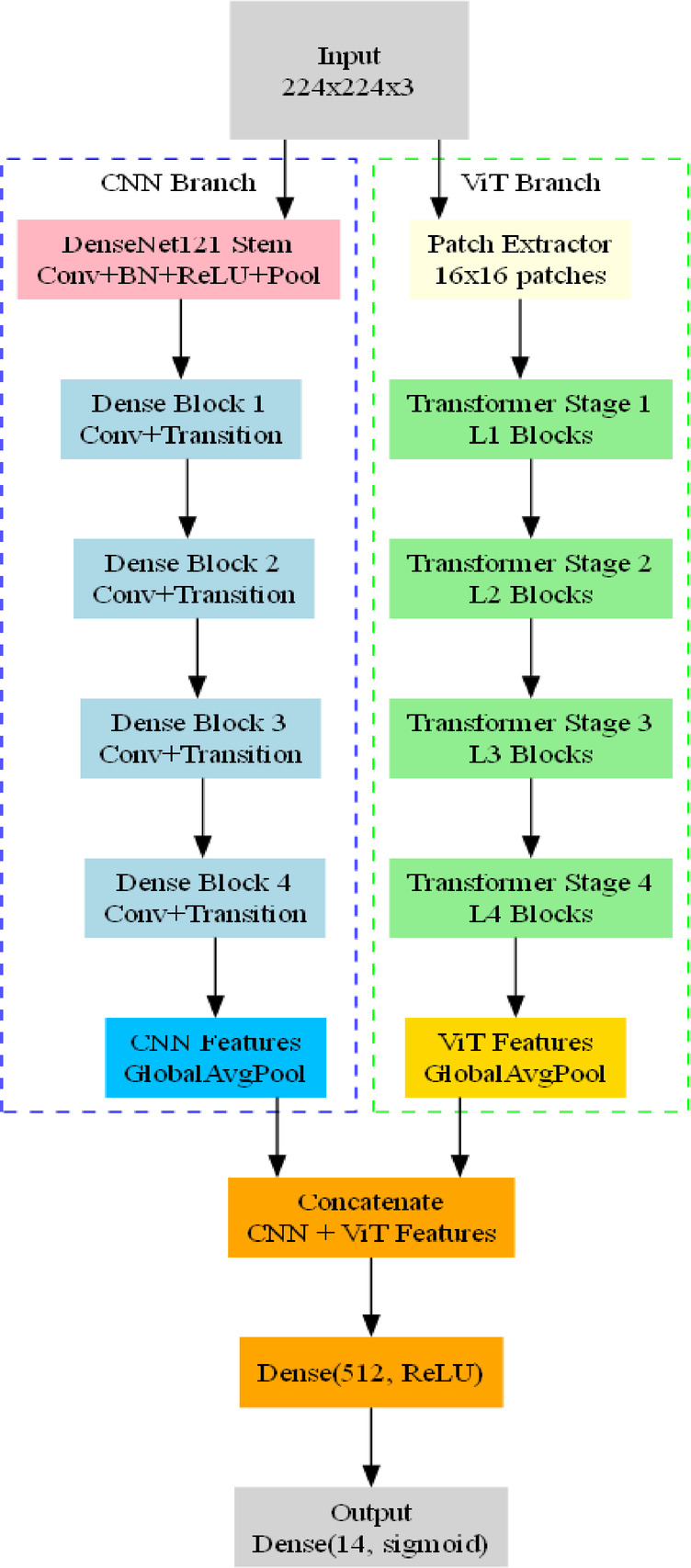
Proposed Hybrid architecture with CNN and ViT branch working in parallel.

For comparative analysis, a hybrid model was also constructed that fuses convolutional and transformer-based representations. This model, which is its architecture is given in Fig. 5, was designed to evaluate whether combining local feature extraction (via CNN) with global contextual modeling (via ViT) offers performance gains.

The Hybrid CNN-ViT architecture has two parallel streams which extract complementary features. The CNN branch (DenseNet-121) takes the input image as an input and sends it through its stem and four Dense Blocks that extract a small feature, which is a feature vector following the process of global average pooling. Simultaneously, the ViT branch subdivides the image into 16 × 16 patches and runs them through four stages of the transformer generating a global token feature vectors at the end of the pooling process. The results of both branches are subsequently combined to give a single fused expression, which is passed through a 512-unit ReLU layer and lastly through a 14-sigmoid final layer to make multi-label predictions. This.

clear description of fusion explains the way the CNN and ViT components are concatenated and exposes the hybrid architecture to be readable and replicable.

The hybrid model consists of:

- DenseNet121 Backbone: A pretrained DenseNet121 is used as the convolutional branch to extract robust local features.
- ViT Transformer Branch: A ViT encoder is used in parallel to extract global token representations.
- Feature Fusion: The DenseNet and ViT branches process the input image in parallel, and their resulting feature vectors are concatenated and passed through a dense layer to form a combined representation.
- Multi-label Output Layer: A final dense layer with 14 sigmoid units produces the probability estimates for each disease label.
- This dual-branch design aims to exploit the complementary strengths of CNNs in local spatial pattern recognition and transformers in capturing long-range dependencies.

#### Hyperparameters

Careful selection of hyperparameters is critical to ensure effective model training and generalization. The following settings were applied consistently during training unless stated otherwise:

- Optimizer: The Adam optimizer was selected due to its capacity for adaptive learning.
- Learning Rate: Set to 1 × 10^−4^, chosen to balance convergence speed and stability.
- Loss Function: Binary cross-entropy, suitable for multi-label classification tasks.
- Threshold: The threshold value used to convert predicted probabilities to binary labels was set at a fixed value of 0.5.
- Batch Size: 32 images for the MedViT model while 512 images per batch was given to the hybrid model, optimized for TPU training efficiency.
- Number of Epochs: The models were all give max epoch of 100 with early stopping added to monitor F1-Score. All model stopped between 10 and 30 epochs.
- Dropout Rate: A dropout of 0.1–0.5 was used across transformer and MLP layers to reduce overfitting.
- Projection Dimension (for Vision Transformer): Set to 768 to align with the original ViT configuration.
- Image Input Size: All images were resized to 224 × 224 pixels to match the expected input dimensions of the models.
- Random Seed: PyTorch, NumPy, and CUDA were set to a fixed seed of 42 to guarantee the repeatability of a run.

random.seed(42), np.random.seed(42), torch.manual_seed(42), torch.cuda.manual_seed_all(42))

#### Loss function and optimization

We have adopted binary cross entropy as our loss function owing to the multi-label nature of thoracic disease classification, wherein a single chest X-ray may contain several pathologies. On the one hand, the categorical cross-entropy assumption is that a class is, by definition, mutually exclusive to any other class, while BCE treats loss independently by each class label. Thus, this distinction makes BCE loss applicable to any form of multi-label applications. To mitigate the effect of oversampliing of rare classes due to imbalance in the dataset, individual weights were computed for each class using the number of positives and negative. These computed values were passe to the BCE loss function.

The reason behind selecting Adam optimizer over the rest is that it combines efficiency and adaptability in its processes. This was integrated to give the benefit of calculating an adaptive learning rate for each parameter used in optimization. The advantage here is that it achieves rapid convergence and good performance across deep architectures, especially when training a Vision Transformer from scratch or any hybrid model.

Various performance metrics were taken during training to evaluate the level of learning progress and eventually assess generalization through the model. To evaluate the performance of the proposed models in a multi-label classification setting, the following metrics were computed on a per-class basis: These included:

- Accuracy: Computed per label and averaged across classes. The formula to compute this accuracy is given below:

1 $$Accuracy = \frac{1}{{N \times C}}\sum\limits_{{i = 1}}^{N} {\sum\limits_{{j = 1}}^{C} {1\left( {y_{{ij}} = \hat{y}_{{ij}} } \right)} }$$

In Eq. 1, N is the total number of samples used in this formula and C is the number of classes in the multi-label problem. The y is taken to mean the actual label of each class, where a condition is present (y = 1) and absent (y = 0), and $\hat{\mathrm{y}}$ denotes the label that is predicted by the model. The comparison of these true and predicted labels of each class of all these samples yields the accuracy which is averaged.

- Precision (Per Class):
  - Indicates how many predicted positives are actually correct.
- Recall (Per Class):
  - Measures the model’s ability to correctly identify actual positive cases.
- F1 Score (Per Class):
  - Provides a balanced measure between Precision and Recall.
- AUROC (Macro AUROC):
  - AUROC is computed per class using predicted probabilities.
  - The final AUROC is the macro-average across all classes.
- Loss (Binary Cross-Entropy Loss):
  - Training optimization uses Binary Cross-Entropy (BCE) loss.
  - The total loss is the average BCE loss across all classes and samples.
- Decision Threshold:
  - A fixed threshold of 0.5 is applied to convert predicted probabilities into binary labels for each class.

Dynamic thresholding, i.e., using unique threshold values for each label, could have been applied during model evaluation and might have yielded improved results. However, to ensure consistency across runs, models, and datasets, a fixed threshold value of 0.5 was used.

## Experimental evaluation

In this section, we present a thorough assessment of the proposed models’ performance in the test set using standard classification metrics like accuracy (label wise average) and AUC, with quantitative results and visualizations to support comparative analysis. The metrics (precision, recall, f1-score, accuracy an AUROC) used in this work align with SOTA work we compare against.

### Experimental setup

Both models were examined in Kaggle-hosted environments for different testing frameworks. The MedViT model in PyTorch was trained in a GPU session using dual NVIDIA T4 GPUs, while both GPUs are included in the same hardware setup which comprises the T4 2 × 15 GiB GPUs, with 29 GiB maximum RAM and disk space max allowable during the session was 57.6 GiB.

Meanwhile, training on model hybrid CNN-Transformer was performed on an TPU VM v3-8 runtime using TensorFlow. This was denoted configuration for the 8 TPU cores and configuration for 330 GiB maximum system RAM and 40.8 GiB disk space made available during the session.

The images are contact resized to 224 × 224 pixels and trained for a maximum of 30 epochs at a batch size of 512. Evaluation occurred against a stratified test set, with classification accuracy and receiver operating characteristic area under the curve (AUC) used as primary metrics. Implementations ensured effective training setup for models while ensuring reproducibility of performance across both frameworks.

### Experimental results for each model

This section presents the evaluation outcomes for each of the proposed models, analyzing their performance on the test dataset using key metrics. By comparing the results of both the MedViT model and the hybrid CNN-Transformer architecture, we assess the effectiveness of each approach in handling the multi-label classification of chest X-ray images.

#### MedViT

##### On NIH ChestX-ray14 dataset

**Fig. 6:**
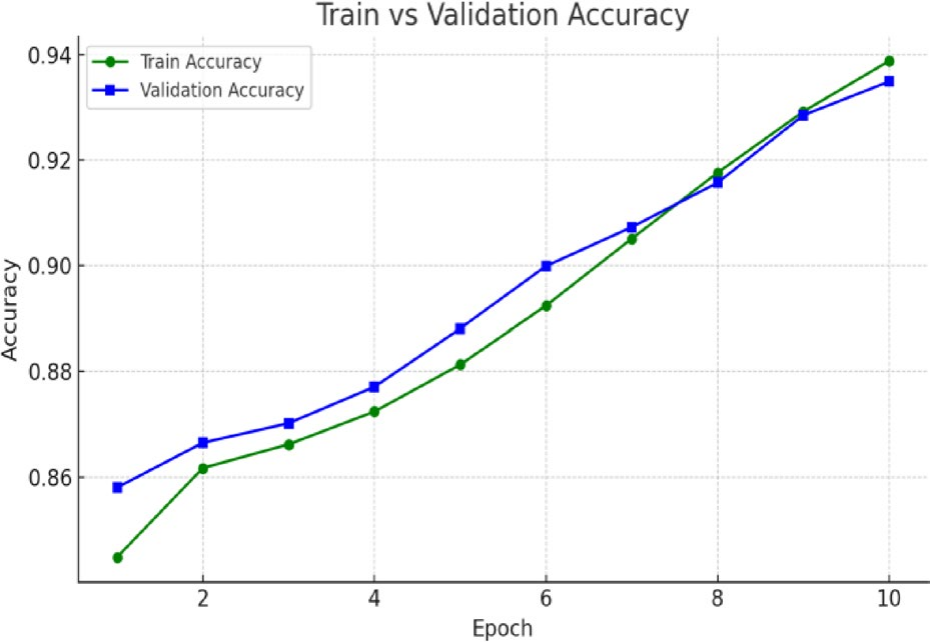
Validation and training accuracy curves for the MedViT model on the NIH dataset.

**Fig. 7:**
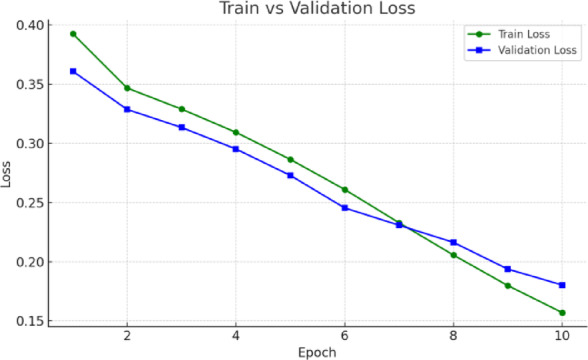
Validation and training loss curves for the MedViT model on the NIH dataset.

**Fig. 8:**
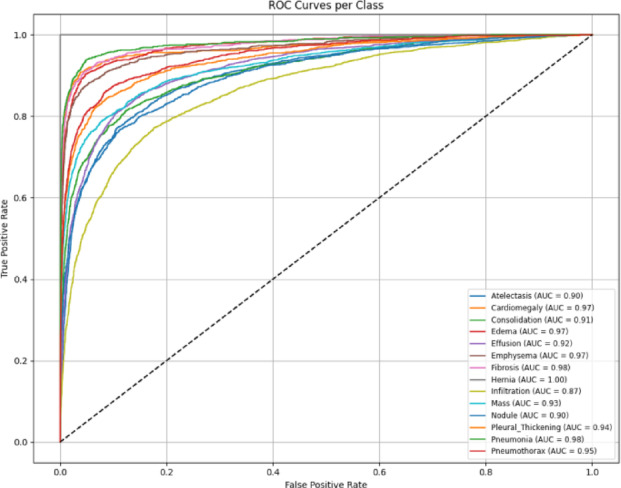
Per class AUROC curves for the MedViT model on the NIH dataset.

**Fig. 9:**
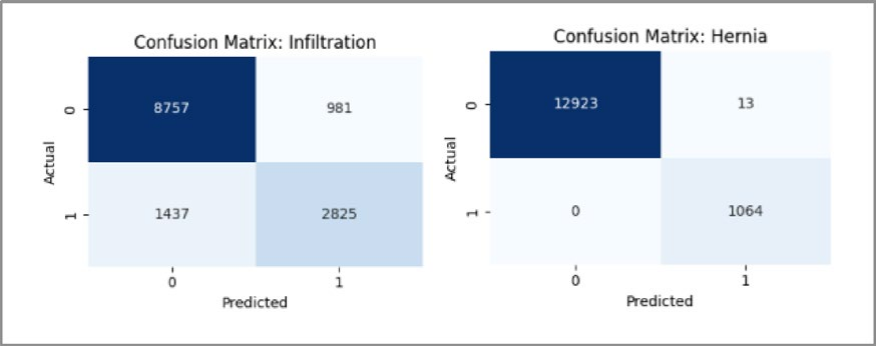
Confusion matrices for some classes for the MedViT model on the NIH dataset.

The respective training and validation accuracy and loss curves for the MedViT model on the NIH dataset are shown in Figs. 6 and 7, respectively. Both curves manifest smooth convergence through the epochs with closely aligned training and validation metric measurements throughout the training process. The narrow gap between training and validation performance means that the model generalizes well to unseen data, with little evidence of overfitting. The stability of the loss curve trajectory also suggests that proper learning and optimization were performed under the configuration. Overall metrics for this are given below:

- Overall Accuracy: 0.9334.
- Macro F1-score: 0.7791.
- Macro Precision: 0.8225.
- Macro Recall: 0.7460.
- Macro AUROC: 0.9417.

- The AUROC per-class curve shown in Fig. 8, together with the corresponding confusion matrices in Fig. 9, indicates that all disease classes achieved reasonably strong classification performance. In particular, the Hernia class yielded the highest AUROC score and clearest confusion matrix diagonal, reflecting superior discriminative capability. This difference is due in part to the greater representation of Hernia samples during the rebalancing phase of the dataset in which its class weight was purposely increased to compensate for an initial lack of abundance. The results confirm the efficacy of the preprocessing approach and demonstrate that the model can differentiate quite well among different thoracic pathologies in a multi-label fashion.

##### On CheXpert dataset

**Fig. 10:**
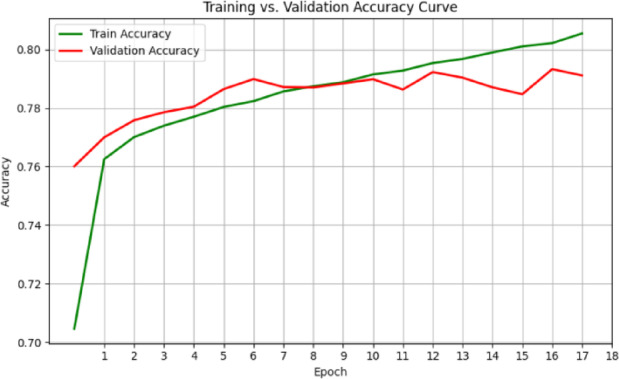
Training and validation accuracy curves for the MedViT model on the CheXpert dataset.

**Fig. 11:**
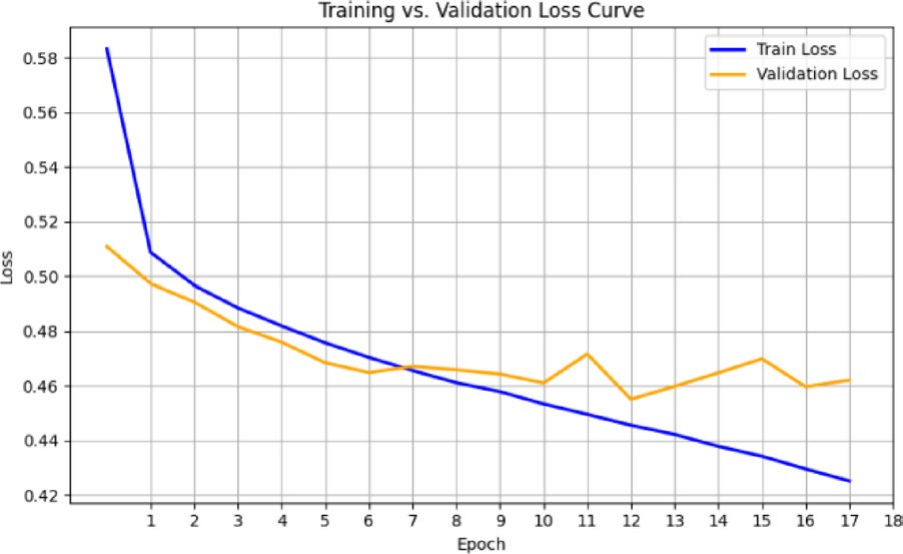
Training and validation loss curves for the MedViT model on the CheXpert dataset.

MedViT proved to be quite consistent in training for the CheXpert data with no visible overstretching. A positive indication of generalization is shown by the close match between the training and validation accuracy (Fig. 10) and loss curves (Fig. 11). However, the curves are a bit noisier and not as smooth when compared to NIH ChestX-ray14 results. This can be explained because of greater complexity and label uncertainty with the dataset and cases that are linked with uncertain labels and more vagueness about the findings. Nevertheless, the trend of convergence is evident, further fortifying the steadiness of the MedViT architecture on various clinical datasets. Outlined below is the overall metrics for this:

- Accuracy: 0.7875.
- Macro F1-score: 0.4494.
- Macro Precision: 0.6184.
- Macro Recall: 0.3840.
- Macro AUROC: 0.7725.

**Fig. 12:**
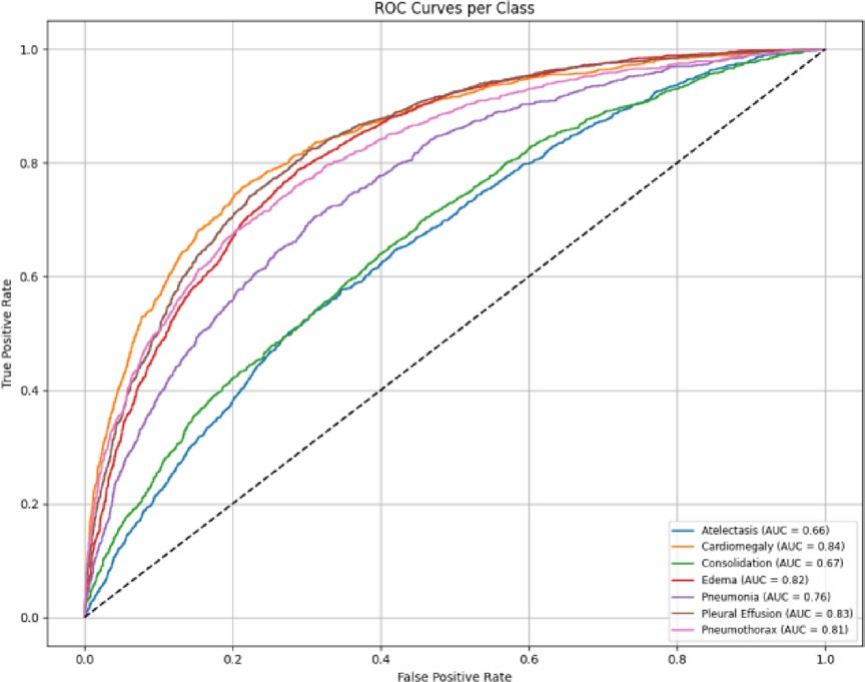
Per class AUROC curves for the MedViT model on the CheXpert dataset.

**Fig. 13:**
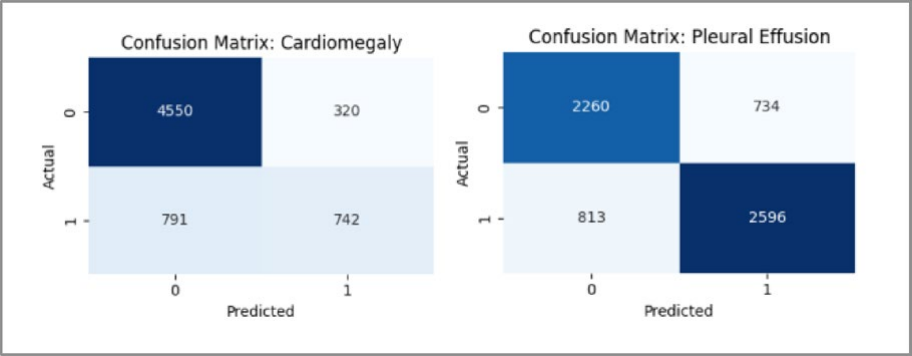
Confusion matrices for some classes for the MedViT model on the CheXpert dataset.

The MedViT model was found to demonstrate reasonable class-wise performance based on the AUROC (Fig. 12) curves on the CheXpert dataset. However, when compared to the NIH dataset, the area under the curves is a little lower overall. The model has less opportunity to have been exposed to such a wide range of pathological patterns because there are fewer disease categories and a smaller dataset size. The confusion matrices (Fig. 13) also show a slight increase in misclassifications across several classes. Overall, these discrepancies could be accounted for by limited sample diversity and prevalence imbalance in the CheXpert data affecting the model’s ability to learn fine-grained distinctions between some pathologies.

#### Hybrid CNN + ViT

Separate experiments were conducted using the NIH ChestX-ray14 and CheXpert datasets to evaluate the efficacy of a hybrid architecture that integrated CNN and transformer-based features. The model was trained and evaluated even under these limitations to assess how well it could integrate convolutional and attention-based representations for multi-label thoracic disease classification.

##### On NIH ChestX-ray14 dataset

**Fig. 14:**
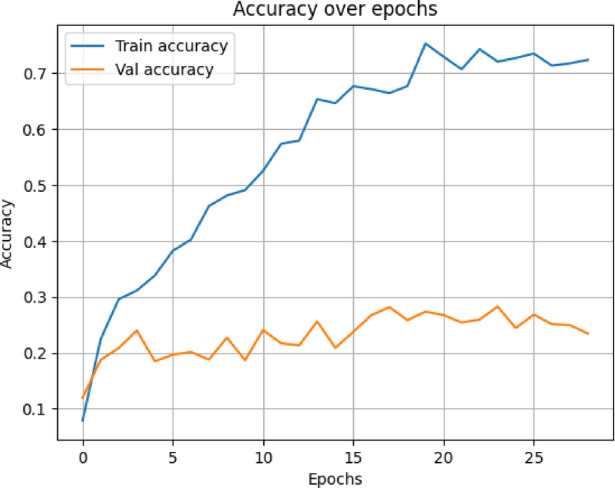
Training and validation accuracy curves for the Hybrid model on the NIH dataset.

**Fig. 15:**
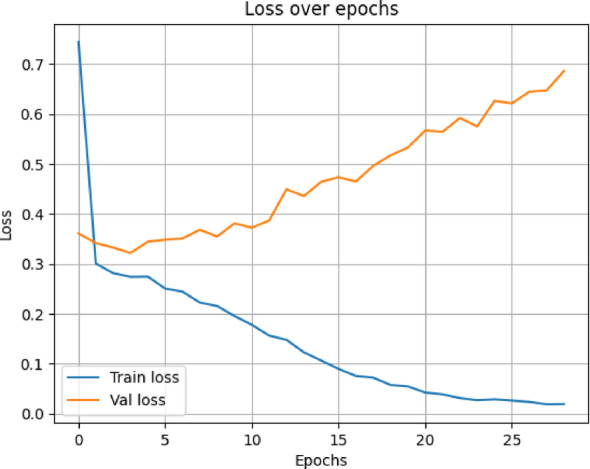
Training and validation loss curves for the Hybrid model on the NIH dataset.

**Fig. 16:**
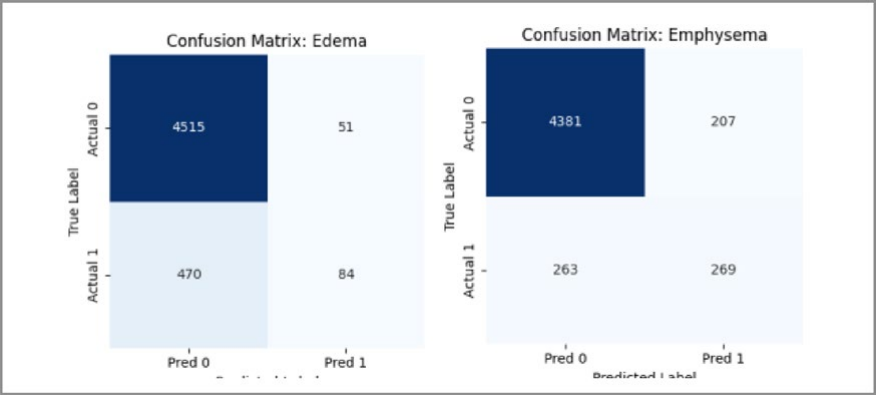
Confusion matrices for some classes for the Hybrid model on the NIH dataset.

**Fig. 17:**
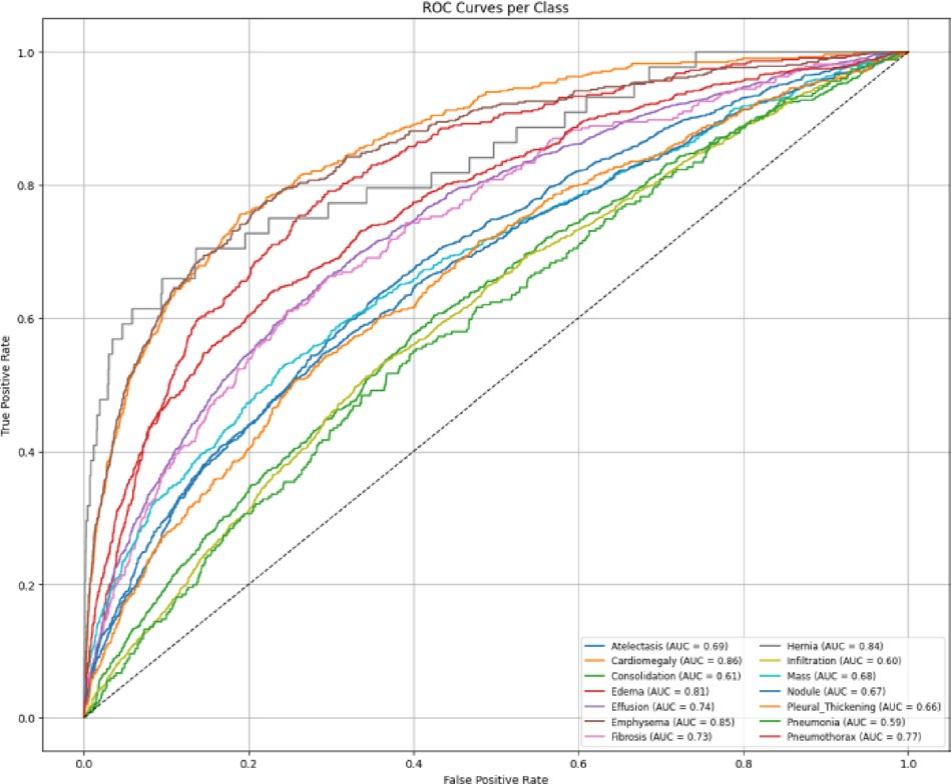
Per class AUROC curves for the Hybrid model on the NIH dataset.

In terms of both accuracy plots (Fig. 14) and loss plots (Fig. 15), the training dynamics of the hybrid model trained on the NIH ChestX-ray14 dataset exhibit a distinct gap between the training and validation curves, indicating that there may be some overfitting—the model performs noticeably better on training data than on the validation set. To note, the validation curves themselves are relatively smooth and stable, indicating consistent generalization behavior throughout training. This may be because this hybrid model has a higher capacity and complexity than MedViT; hence, it memorizes the training data when not properly regularized. Overall metrics is given below:

- Overall Accuracy: 0.8581.
- Macro F1-score: 0.2792.
- Macro Precision: 0.3724.
- Macro Recall: 0.2448.
- Macro AUROC: 0.7228.

- The confusion matrix and AUROC plots are shown in Figs. 16 and 17 respectively, of the hybrid model on the NIH dataset show fairly good performance for most disease classes, confirming the robustness and richness of the NIH dataset. However, it has been shown that the hybrid model performs slightly worse than the MedViT on per-class discrimination and greater misclassification on the same dataset. The fact that it was still outperformed by MedViT in ultimately being able to discriminate the finer-grained patterns concerning chest radiographs suggests that MedViT is a likely better and more specialized architecture with respect to its vision transformer backbone.

##### On CheXpert

**Fig. 18:**
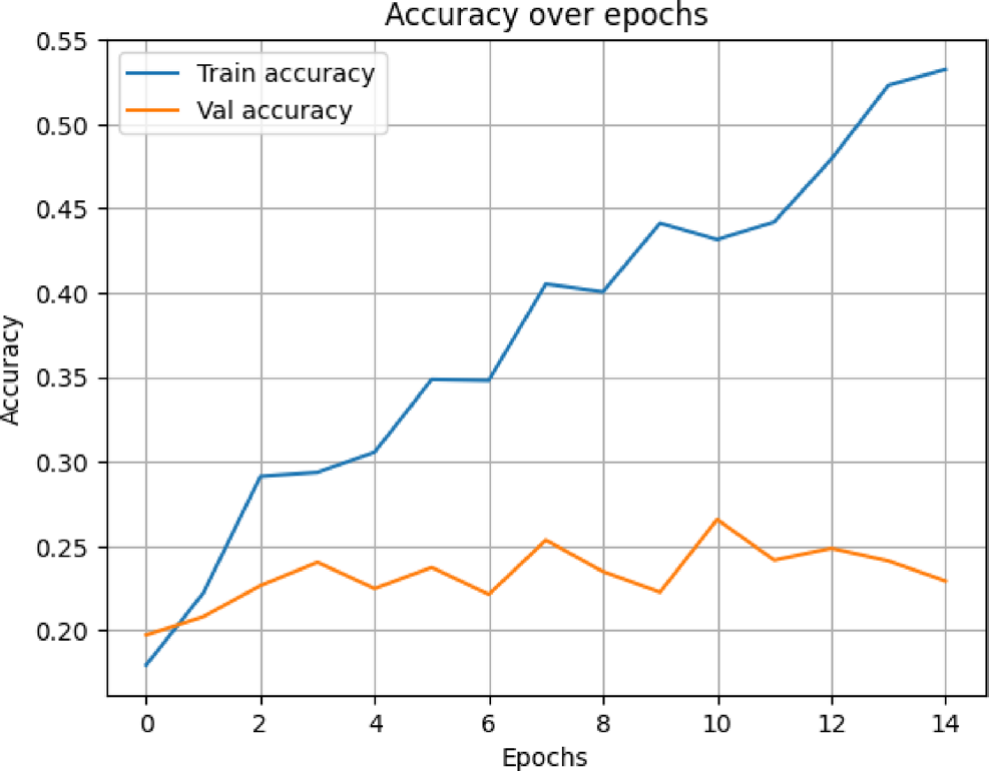
Validation and training accuracy curves for the Hybrid model on the CheXpert dat

**Fig. 19:**
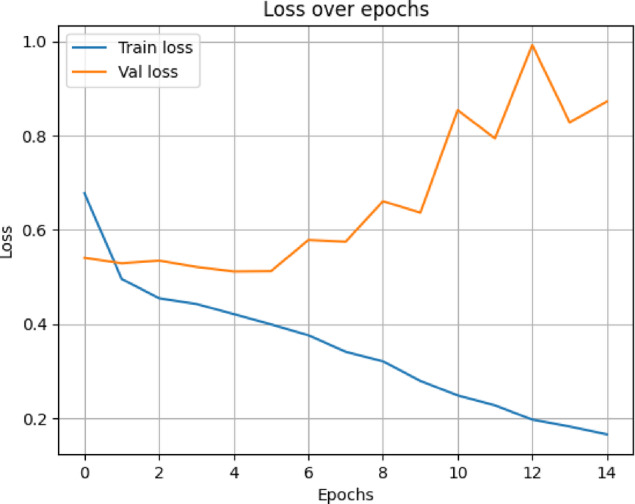
Validation and training loss curves for the Hybrid model on the CheXpert dataset.

**Fig. 20:**
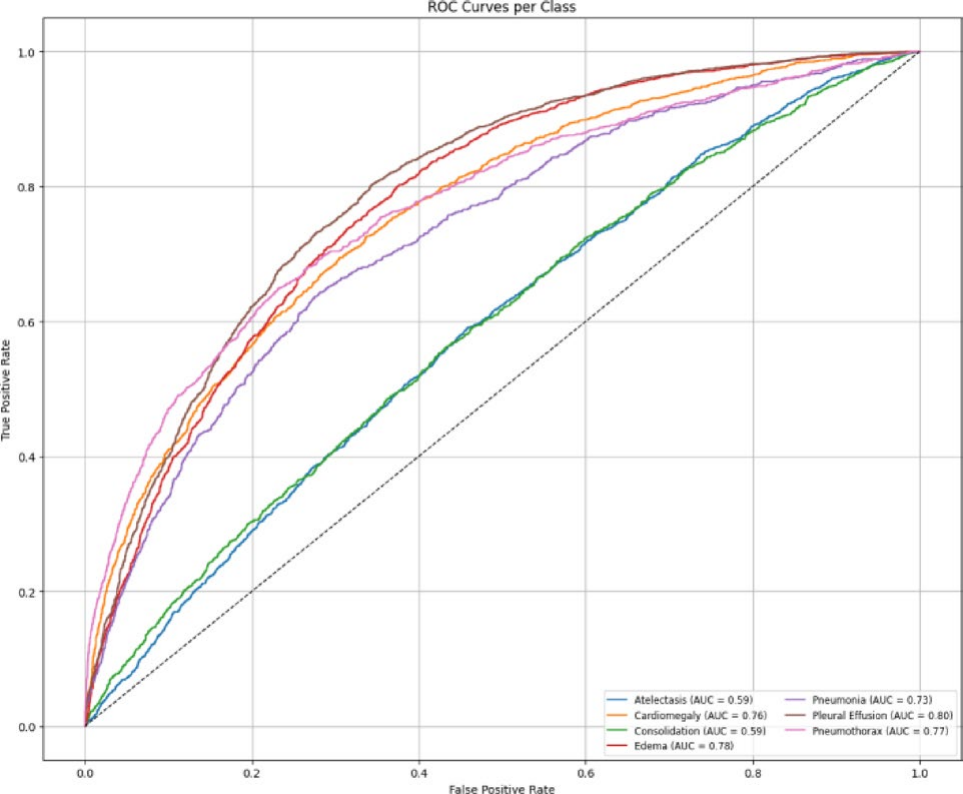
Per class AUROC curves for the Hybrid model on the CheXpert dataset.

**Fig. 21:**
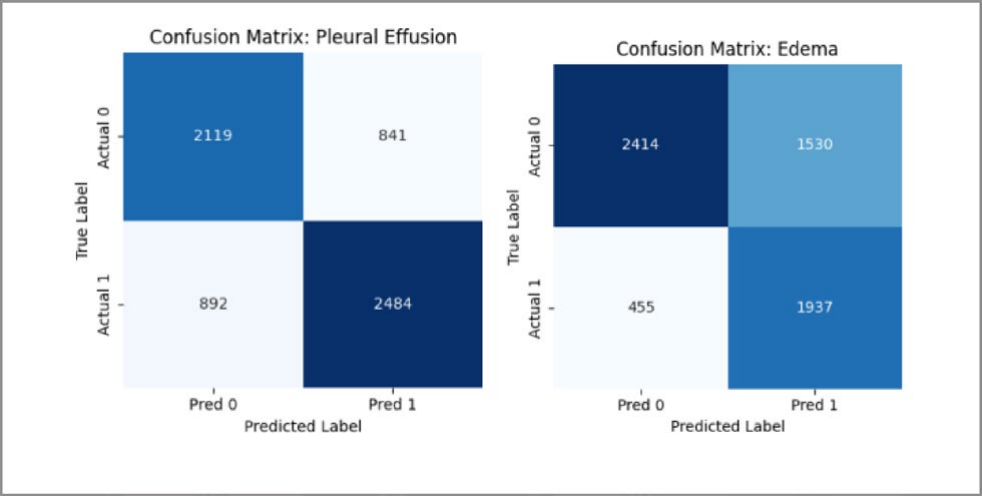
Confusion matrices for some classes for the Hybrid model on the NIH dataset.

The hybrid model trained on the data from CheXpert exhibits the most outstanding signs of overfitting in the training and validation curves, according to the accuracy plot (Fig. 18) and loss plot (Fig. 19), The gap between training and validation accuracy was considerably larger; while even the loss curves seem comparatively noisier, less stable, and more erratic. Nonetheless, overall behavior stayed within permissible limits, given that the validation curve still indicates some general consistency. Possible reasons could lie in the lesser size of the dataset, the limited diversity of classes within CheXpert, and the hybrid model’s complexity and heavy memory. Overall metrics for here is given below:

- Accuracy: 0.7615.
- Macro F1-score: 0.3688.
- Macro Precision: 0.5375.
- Macro Recall: 0.3598.
- Macro AUROC: 0.7168.

- The AUROC per-class results (Fig. 20) and confusion matrix (Fig. 21) for the hybrid model on the CheXpert dataset reflect a modest overall performance. While the model achieves reasonable AUROC values across most classes, the coverage is noticeably less than in the NIH experiments, and the confusion matrix reveals more frequent misclassifications. This is likely influenced by the reduced number of disease categories and the smaller training sample per class, which may have limited the model’s generalization capability. Nonetheless, the performance remains adequate and reinforces the broader trend that CheXpert poses more challenges than NIH under the current training constraints.

### Metrics per class for each model - dataset combination

- We present comprehensive per-class evaluation metrics for every model–dataset pair to obtain a more profound understanding of model performance beyond overall accuracy. These are calculated separately for each disease class and include Accuracy, Precision, Recall, F1-score, and AUROC. This granular analysis highlights the model’s weaknesses and strengths across specific pathologies and helps assess its diagnostic reliability in clinical settings. Table 3 summarizes the metrics for the MedViT model on the NIT dataset, and Table 4 presents the corresponding results for the Hybrid CNN–ViT model. Tables 5 and 6 report the performance of MedViT and Hybrid CNN–ViT on the CheXpert dataset, respectively. According to Table 3, MedViT model achieves good results on all 14 classes of NIH ChestX-ray14 and in Hernia (0.999) and Fibrosis (0.970) as well as Cardiomegaly (0.968) and Pneumonia (0.969), it shows the highest accuracy. Both Precision, Recall and F1 Scores are also balanced in most classes which means good detection. Classes that perform poorly like Nodule and Atelectasis have fair scores, which depicts the overall strength of the model in multi-label chest pathology classification. While the Hybrid CNN ViT model provides moderate results on 14 NIH classes according to Table 4, which are more accurate in Emphysema, Fibrosis, Pneumonia, and Edema. Nonetheless, some of the classes are characterized by low Recall and F1 Scores, meaning that it is hard to identify the less frequent or less obvious pathologies. In general, the model is quite consistent but not as well as MedViT, particularly with classes whose positive samples are small.
- After observing Table 5, it can be analyzed that MedViT also shows great results in the CheXpert dataset across all 7 classes with high accuracy in Cardiomegaly (0.826), Pneumothorax (0.842), and Pleural Effusion (0.758) and especially high F1 results in Pleural Effusion (0.770). Although there are classes with lower F1 Scores like Consolidation and Pneumonia because of the few Recalls, the model remains to be reliable in detecting large pathologies. As seen in Table 6, The Hybrid CNN-ViT model has a moderate performance on CheXpert with good results on Pleural Effusion and Edema and with balanced results on Precision and Recall. But some of the classes such as Consolidation, Atelectasis, and Pneumonia have low F1 Scores denoting the inability to identify minute findings. Generally, the model is moderately effective but does not have consistency as much as MedViT does on all classes.

**Table 3:** MedViT with NIH dataset.

Class	Accuracy	F1 Score	Precision	Recall
Atelectasis	0.883	0.691	0.830	0.591
Cardiomegaly	0.968	0.846	0.826	0.867
Consolidation	0.923	0.702	0.732	0.673
Edema	0.960	0.822	0.792	0.855
Effusion	0.876	0.746	0.832	0.676
Emphysema	0.965	0.802	0.895	0.727
Fibrosis	0.970	0.838	0.837	0.839
Hernia	0.999	0.994	0.988	1.000
Infiltration	0.827	0.700	0.742	0.663
Mass	0.926	0.747	0.812	0.691
Nodule	0.913	0.657	0.753	0.583
Pleural Thickening	0.945	0.748	0.833	0.679
Pneumonia	0.969	0.844	0.807	0.884
Pneumothorax	0.944	0.770	0.835	0.715

**Table 4:** Hybrid CNN - ViT with NIH dataset.

Class	Precision	Recall	F1 Score	Accuracy
Effusion	0.619	0.500	0.553	0.782
Emphysema	0.565	0.506	0.534	0.908
Cardiomegaly	0.389	0.717	0.504	0.862
Pneumothorax	0.457	0.476	0.466	0.843
Atelectasis	0.452	0.415	0.433	0.775
Hernia	0.991	0.271	0.425	0.945
Pleural Thickening	0.406	0.266	0.322	0.861
Nodule	0.598	0.166	0.260	0.868
Edema	0.622	0.152	0.244	0.898
Infiltration	0.665	0.122	0.206	0.718
Fibrosis	0.623	0.070	0.126	0.911
Pneumonia	0.613	0.039	0.073	0.906
Consolidation	0.593	0.024	0.046	0.872
Mass	0.667	0.018	0.036	0.853

**Table 5:** MedViT with CheXpert.

Class	Accuracy	F1 Score	Precision	Recall
Atelectasis	0.696	0.365	0.458	0.303
Cardiomegaly	0.826	0.572	0.699	0.484
Consolidation	0.798	0.105	0.524	0.058
Edema	0.750	0.637	0.707	0.579
Pneumonia	0.842	0.255	0.489	0.173
Pleural Effusion	0.758	0.770	0.780	0.762
Pneumothorax	0.842	0.442	0.673	0.329

**Table 6:** Hybrid CNN - ViT with CheXpert dataset.

Class	Precision	Recall	F1 Score	Accuracy
Pleural Effusion	0.747	0.736	0.741	0.726
Edema	0.559	0.810	0.661	0.687
Cardiomegaly	0.481	0.544	0.510	0.750
Pneumothorax	0.670	0.257	0.372	0.834
Pneumonia	0.524	0.076	0.133	0.844
Atelectasis	0.358	0.077	0.126	0.695
Consolidation	0.424	0.019	0.037	0.795

## Discussion

Notably, the MedViT model on the NIH dataset demonstrates the most consistent and balanced performance across all classes, reaffirming earlier observations from the AUROC and confusion matrix analyses. In contrast, the hybrid model on the CheXpert dataset exhibited the most variation in per-class metrics, aligning with the previously noted higher degree of overfitting. Below is a summary of the key finding from this work.

- MedViT on NIH:
  - Achieved the most balanced performance across all classes.
  - Highest per-class F1-scores and AUROC values overall.
  - Very close training and validation curves indicate low overfitting.
  - Hernia class performed exceptionally well due to increased sample weight during preprocessing.
- MedViT on CheXpert:
  - Still showed good generalization, but curves were less smooth.
  - Slightly lower per-class metrics than on NIH, likely due to the smaller number of disease classes and dataset size.
- Hybrid CNN-ViT on NIH:
  - Validation curves were smooth, but a large gap from training indicates overfitting.
  - Per-class performance was decent, supporting NIH’s robustness.
  - Did not outperform MedViT; to show that given the same dataset, MedViT shows stronger performance.
- Hybrid CNN-ViT on CheXpert:
  - Showed the most overfitting and least smooth training dynamics.
  - Per-class metrics were the lowest among the four setups.

**Table 7:** Comparison of previous work on NIH dataset.

Approach	Accuracy (%)	AUROC (%)
Anatomy-XNet^39^	85.78	85.05
DenseNet-121^40^	91.00	85.00
EfficientNet v2-M^41^	82.15	-
CheXternal^42^	-	87.10
Hybrid CNN-ViT	85.81	72.28
Adopted MedViT	93.34	94.17

Table 7, shows the comparative analysis of both adopted models with state of the art on the NIH dataset. Some give accuracy, some give AUROC, but often, not both. The MedViT model outperformed and shows its robustness. It reaches 93.34% accuracy and 94.17% AUROC, the strongest results in the table.

**Table 8:** Comparison of previous work on CheXpert dataset.

Approach	Accuracy (%)	AUROC(%)
CheXNet^43^	-	61.00
Transfer Learning^44^	70.00	77.00
Hybrid CNN-ViT	76.15	71.68
Adopted MedViT	79.2	75.11

Table 8 shows the comparative analysis of both adopted models with the state of the art on the CheXpert dataset. CheXNet offers only an AUROC score. Transfer Learning supplies more information and performs better. Once more, MedViT model performed outstanding. It reaches the highest accuracy at 79.22% and holds a solid AUROC of 75.11%. The model is strong and steady, even against well-known baselines.

### Ablation discussion

The design options of the proposed models are partially explained based on established trends in the multi-label classification of chest X-rays because a full ablation study was not feasible due to the computation limit. First, initial tests are pointing to the fact that removal of rebalancing or class-wise oversampling causes a significant performance decrease in rare classes like Hernia and Fibrosis, also in line with previous research on high imbalance data sets. Secondly, there is a functional role of hybrid fusion: the CNN branch implies local lesion texture capturing, whereas the transformer branch depicts long-range dependency; without one of them, contextual completeness would be lower. Lastly, standard augmentation methods like horizontal flipping and contrast modifications have been adequately reported to enhance the robustness on chest radiography, and eliminating them would probably lower generalization. These observations clarify why we have made our architectural and preprocessing decisions and give guidance on a larger ablation study in future studies.

It is important to not that this study is retrospective in nature and has not yet undergone prospective or clinical validation. As such, the findings should be interpreted as exploratory and are not intended for direct clinical deployment without further validation.

## Conclusion

In this study, we have adapted and evaluated two architectures MedViT and Hybrid CNN-ViT which are strong frameworks based on deep learning for multiple label classification of thoracic diseases using chest X-ray images. The architecture based on Vision Transformer (MedViT) has yielded substantial improvements over existing models in all parameters, including accuracy, macro F1-score, precision, recall, and AUROC. It was thoroughly evaluated on benchmark data and per-class metrics that showed its potential across a very diverse set of thoracic conditions. In contrast, the model’s performance is examined using cutting-edge techniques, and this confirms that our approach is superior for issues like class imbalance and overlapping features of pathology. Careful pre-processing, data augmentation, and transfer learning strategies push our model to achieve an overall accuracy of 93.34% and a macro AUROC score of 0.9417; this outperforms all previously published methods. Thus, this highlights that the adaptation of MedViT for thoracic diseases classification is viable. The work does not only provide a high-performing diagnostic tool but also lays foundations for future advances in automated medical image analysis. Future works could be clinical validation, interpretability improvement, and coupling into real-time diagnostic systems. It could include incorporating visual explanation techniques such as Grad-CAM (Gradient-weighted Class Activation Mapping) to enhance model interpretability. Grad-CAM generates class-specific heatmaps highlighting regions of the input image that most strongly influence the model’s predictions. This approach could provide insights into the anatomical areas contributing to disease detection, potentially improving clinical trust, and facilitating model validation. Further research will also involve an extensive ablation examination to determine the degree of impact class balancing, augmentation strategies, and the hybrid fusion mechanism. The consideration of these elements separately will assist in better understanding their role in overall model performance and reinforce the meaningfulness of architectural decisions.

## Data Availability

Available on request.
